# The effects of Sodium-glucose cotransporter 2 inhibitors on adipose tissue in patients with type 2 diabetes: A meta-analysis of randomized controlled trials

**DOI:** 10.3389/fendo.2023.1115321

**Published:** 2023-01-27

**Authors:** Xindong Liu, Ying Chen, Tao Liu, Ling Cai, Xiaofeng Yang, Chuan Mou

**Affiliations:** Department of Cardiovascular Medicine, Nanchong Central Hospital, The Second Clinical Medical College of North Sichuan Medical College, Nanchong, China

**Keywords:** Sodium-glucose cotransporter 2 inhibitors, SGLT2, type 2 diabetes, adipose tissue, meta-analysis

## Abstract

**Purpose:**

To systematically evaluate the effect of Sodium-glucose cotransporter 2 (SGLT2) inhibitors on adipose tissue in patients with type 2 diabetes.

**Methods:**

We searched PubMed, Cochrane Library, EMBASE, and Web of science databases for literature pertaining to Randomized controlled trials (RCTs) of SGLT2 inhibitors in treating type 2 diabetes patients. The retrieval time was from the date of establishment of the databases to September 1, 2022. Meta-analysis was performed using RevMan5.4 software.

**Results:**

Totally 551 patients were included in 10 articles. Meta-analysis results showed that compared with the control group, the visceral adipose tissue (WMD = -16.29 cm^2^, 95% CI: -25.07 ~ -7.50, P<0.00001), subcutaneous adipose tissue (WMD = -19.34 cm^2^, 95% CI: -36.27 ~ -2.41, P<0.00001), body weight (WMD = -2.36 kg, 95% CI: -2.89 ~ -1.83, P<0.00001) and triglyceride (WMD = -24.41 mg/dl, 95% CI: -45.79 ~ -3.03, P = 0.03) of the trial group significantly reduced.

**Conclusion:**

SGLT2 inhibitors cause significant reductions in visceral adipose tissue, subcutaneous adipose tissue, body weight and triglycerides in type 2 diabetes patients, which may be attributed to the protective effect of the inhibitors on cardiovascular system.

## 1 Introduction

Cardiovascular disease remains one of the leading causes of death worldwide, posing huge health risks and economic burdens. SGLT2 inhibitors are novel hypoglycemic agents that lower blood glucose by inhibiting the reabsorption of glucose in the kidneys and allowing excess glucose to be excreted from the urine. In recent years, a number of large randomized controlled clinical trials (the EMPA­REG OUTCOME trial (1), CANVAS Program (2) and DECLARE­TIMI 58 trial (3), etc.) have confirmed that SGLT2 inhibitors can significantly reduce all-cause mortality, cardiovascular mortality and heart failure hospitalization rates in patients with type 2 diabetes, among others. However, their potential mechanism of cardiovascular protection is not completely clear.

There is considerable evidence that overweight, obesity and their comorbidities increase the morbidity and mortality of cardiovascular disease, independent of age and gender (4–6). A meta-analysis showed that SGLT2 inhibitors significantly reduced body weight in type 2 diabetes patients as compared to placebo (7). Studies using dual-energy X-ray absorptiometry (DEXA) and bioelectrical impedance analysis (BIA) confirmed that the SGLT2 inhibitor-associated weight loss was attributed to the reduction in (viscera and subcutaneous) adipose tissue mass, instead of the lean tissue mass which remained unchanged (8, 9). Type 2 diabetes patients are often accompanied by obesity, and obese type 2 diabetics have a greater propensity for ectopic and visceral fat deposition (10). It has been suggested that SGLT2 inhibitors significantly lowered weight and adiposity indices, with the potential to improve cardiometabolic risk among patients with type 2 diabetes mellitus (11). At present, the effects of SGLT2 inhibitors on visceral adipose tissue (VAT) and subcutaneous adipose tissue (SAT) are uncertain, and large-scale clinical trials are lacking. Therefore, we conducted a meta-analysis to assess the impact of SGLT2 inhibitors on VAT and SAT in patients with type 2 diabetes.

## 2 Methods

The meta-analysis was undertaken in accordance with the recommendations of the Preferred Reporting Items for Systematic Reviews and Meta-Analyses (PRISMA) guidelines (12). The literature search, literature screening, data extraction and risk of bias assessment were carried out independently by two researchers. Any disagreement was resolved by discussion or a third author. All studies included in this paper complied with the Declaration of Helsinki and were approved by the local ethics committee.

### 2.1 Data source and research search strategy

We searched the PubMed, Cochrane Library, EMBASE and Web of science databases from conception to September 1, 2022, for the data from RCTs of SGLT2 inhibitors in treating type 2 diabetes. The main search terms include “Diabetes Mellitus, Type 2”, “Sodium Glucose Transporter 2 Inhibitors”, and “Adipose Tissue”. The detailed information regarding the search strategy is shown in **Supplementary Table 1**. Additionally, we manually searched the references of relevant articles to obtain more literature.

### 2.2 Study selection

Inclusion criteria: 1) articles with type 2 diabetes patients aged ≥ 18 years; 2) trials that used SGLT2 inhibitors in the treatment group and placebo or conventional hypoglycemic drugs in the control group; 3) articles with randomized controlled trial (RCT); and 4) articles with outcome indicators including VAT or SAT that was reported in square centimeters. Exclusion criteria: 1) articles with missing, incomplete or unreasonable trial data; 2) articles with unavailable or converted primary outcome indicators; 3) duplicate literature data; and 4) articles examining animal studies.

### 2.3 Data extraction and assessment of risk of bias

A data extraction form was created to pre-extract information from the eligible literature, and then the data extraction form was further refined. The extracted data included 1) study characteristics (first author, publication date, study design, intervention, follow-up time); 2) study subject characteristics (number of patients, age, gender, weight, body mass index (BMI), duration of diabetes); 3) intervention characteristics (drug name, route of administration, dose, duration of treatment, etc.); 4) primary outcome indicators (visceral adipose tissue area, subcutaneous adipose tissue area and relative measurement methods); and 5) secondary outcome indicators (body weight, BMI, total cholesterol, triglycerides, low density lipoprotein cholesterol (LDL-C), high density lipoprotein cholesterol (HDL-C)). We assessed the treatment-related changes according to the change in the mean values and standard deviations (SD) of the outcome indicators before and after treatment. Detailed information on the changes pre- and post-treatment are shown in **Supplementary Table 2**.

The Cochrane risk-of-bias tool was used to assess the risk of bias based on the following seven criteria: random sequence generation, allocation concealment, blinding of participants and personnel, blinding of outcome assessment, incomplete outcome data, selective reporting, and other bias. Each study was judged to be at ‘low’, ‘high’ or ‘unclear’ risk of bias.

### 2.4 Data synthesis and analysis

All outcome indicators were continuous outcome data, expressed by weighted mean difference (WMD). Meta-analysis was performed using RevMan 5.4 software to calculate combined effect sizes and 95% confidence interval (CI) for each group. Heterogeneity was assessed using I^2^ statistics, with I^2^ of 25-50%, 50%-75% and >75% representing small, moderate and large heterogeneity, respectively. Considering that the heterogeneity among the included studies may be large, all the results are pooled using random effect models. Subgroup analyses were performed according to possible sources of heterogeneity. Statistical descriptions were made using forest plots, and publication bias was assessed by funnel plots. *P*<0.05 was considered statistically significant.

## 3 Results

### 3.1 Study selection

**Figure 1:**
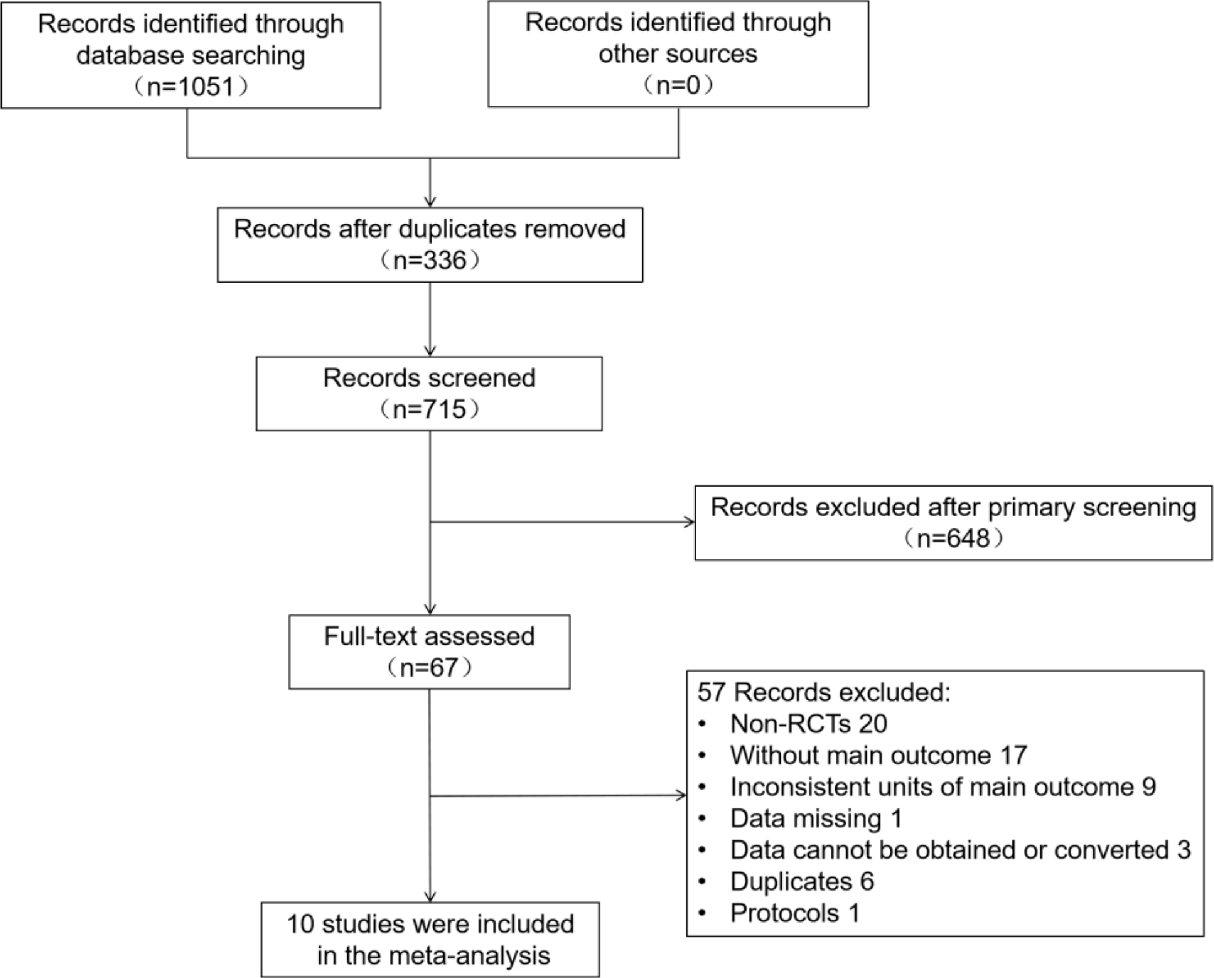
Flow chart of literature screening.

**Figure 1** illustrates the literature screening process. A total of 1051 articles were obtained after searching the databases based on the search strategy. The specific databases and corresponding literature quantity were as follows: PubMed (134), Cochrane Library (227), EMBASE (466), Web of science (224). 336 duplicates were removed by using Noteexpress software, 67 articles remained after the initial screening by reading the abstracts and full text. After excluding non-RCTs, trials failing to comply with study design protocols, trials lacking main outcome indicators, articles with missing data, and duplicate literature, 10 studies were included in the final meta-analysis (9, 13–21). 2 literature were sub-studies (13, 14), some research data of which were obtained from their original studies (22, 23).

### 3.2 Characteristics of the included studies

The baselines of all the included studies are comparable between groups. **Table 1** shows the characteristics of the included studies. A total of 551 patients were enrolled, including 306 in the intervention group and 245 in the control group. Three SGLT2 inhibitors were evaluated in the studies, namely empagliflozin, dapagliflozin and ipragliflozin. The follow-up time was 3 or 6 months for most studies, and 26 months for only one study.

**Table 1:** Characteristics of the included studies.

Study	Age (y)	BMI (kg/m^2^)	Diabetes duration (y)	Follow-up (weeks)	Number of patients	Intervention	Instrument
Kim 2014 (13)	55.7 ± 10.4	31.9 ± 4.9	/	104	29	empagliflozin 25mg qd + metformin	MRI
					22	glimepiride 1-4mg qd + metformin	
Bando 2017 (14)	55.0 ± 8.6	27.6 ± 3.6	9.7 ± 4.4	12	40	ipragliflozin 50 mg qd	CT
					22	continued previous treatment	
Ito 2017 (15)	58.2 ± 10.9	30.3 ± 5.6	9.1 ± 5.8	24	32	ipragliflozin 50 mg qd	CT
					34	pioglitazone 15–30 mg qd	
Inoue 2019 (9)	60.7 ± 10.9	27.8 ± 4.2	17. 5± 9.4	24	24	ipragliflozin 50 mg qd	MRI
					24	continued previous treatment	
Shimizu 2019 (16)	56.6 ± 12.4	27.9 ± 4.2	/	24	33	dapagliflozin 5mg qd	BIA
					24	standard treatment	
Han 2020 (18)	53.9 ± 10.9	30.3 ± 4.6	9.4 ± 5.8	24	30	ipragliflozin 50mg qd + metformin + pioglitazone	CT
					15	metformin + pioglitazone	
Sakurai 2020 (17)	58.6 ± 12.5	27.6 ± 6.0	/	12	31	empagliflozin 10 mg qd	BIA
					18	standard treatment	
Chehrehgosha 2021 (20)	51.2 ± 8.1	30.5 ± 3.9	6.3 ± 4.7	24	35	empagliflozin 10 mg qd	DEXA
					37	placebo	
Gaborit 2021 (19)	56.9 ± 9.6	34.9 ± 6.0	11.1 ± 6.7	12	26	empagliflozin 10 mg qd	MRI
					25	placebo	
Horibe 2022 (21)	60.9 ± 9.7	27.8 ± 3.9	12.5 ± 8.1	24	26	dapagliflozin 5 mg qd	MRI
					24	standard treatment	

CT, computed tomography; MRI, magnetic resonance imaging; DEXA, dual-energy x-ray absorptiometry; BIA, bioelectrical impedance analysis.

### 3.3 Quality assessment

All studies provided methods of random allocation. 3 studies were double-blinded and 7 studies were unblinded trials. One of the articles was a conference literature with incomplete information (13), so its “incomplete outcome data” and “other bias” were classified as unclear risk. Two trials funded by pharmaceutical companies were classified as high risk for ‘other bias’ according to guidance provided by Cochrane (24). Another trial also funded by a pharmaceutical company was classified as low risk for the ‘other bias’ item, because the authors declared that the funder had no role in study design, data collection and analysis, publication decisions or manuscript preparation. **Supplementary Figure 1** provides the quality assessment details.

### 3.4 Meta-analysis of outcomes

#### 3.4.1 VAT and SAT

**Figure 2:**
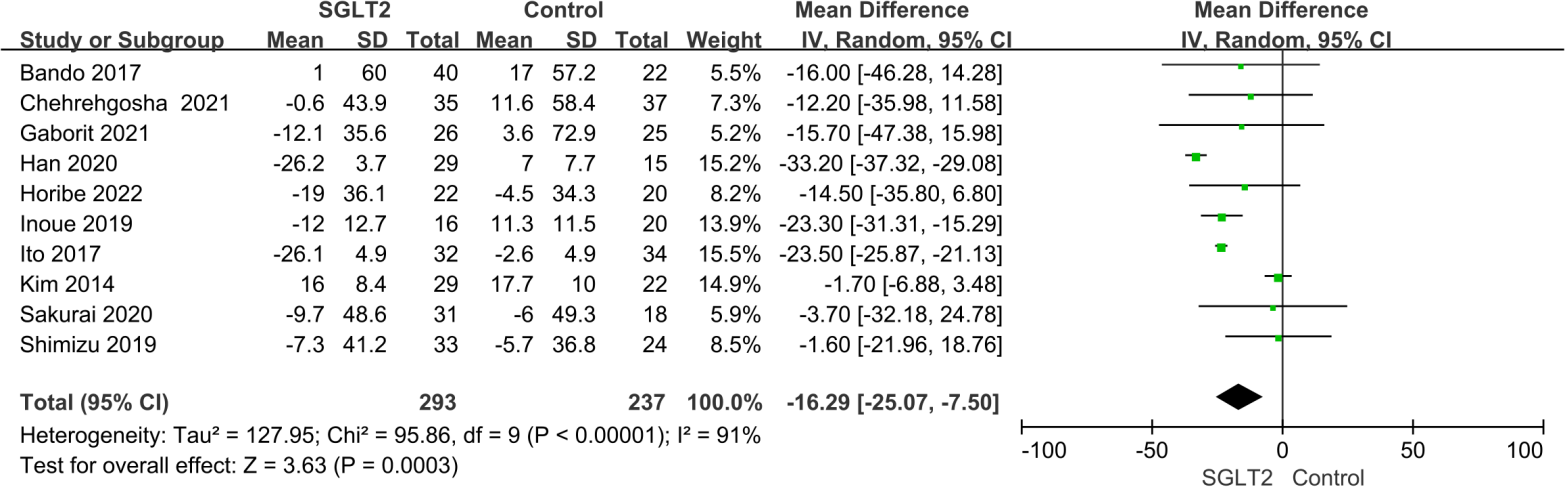
Forest plot of VAT.

**Figure 3:**
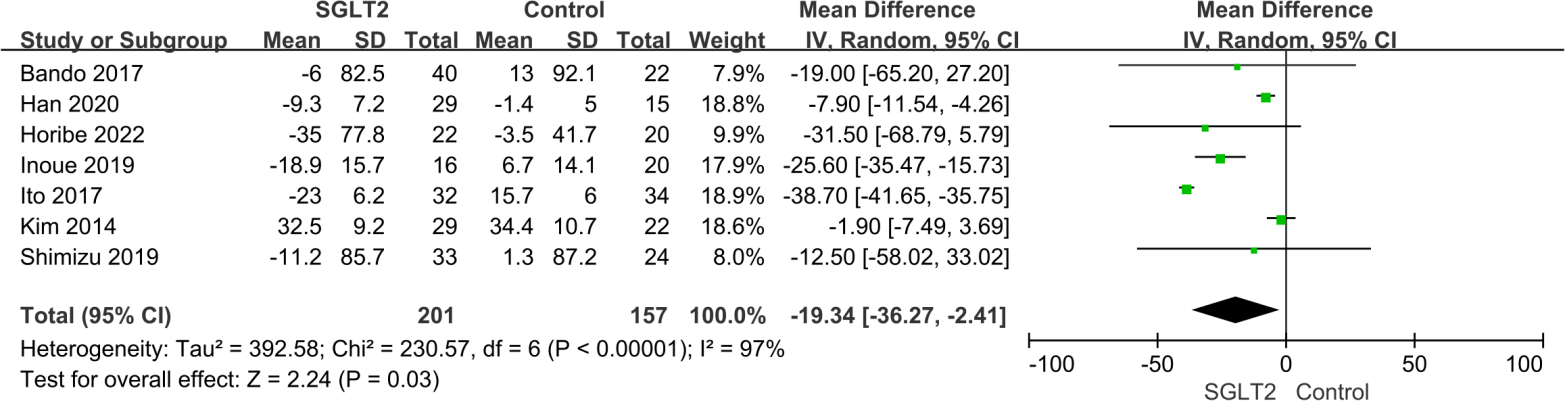
Forest plot of SAT.

**Figure 4:**
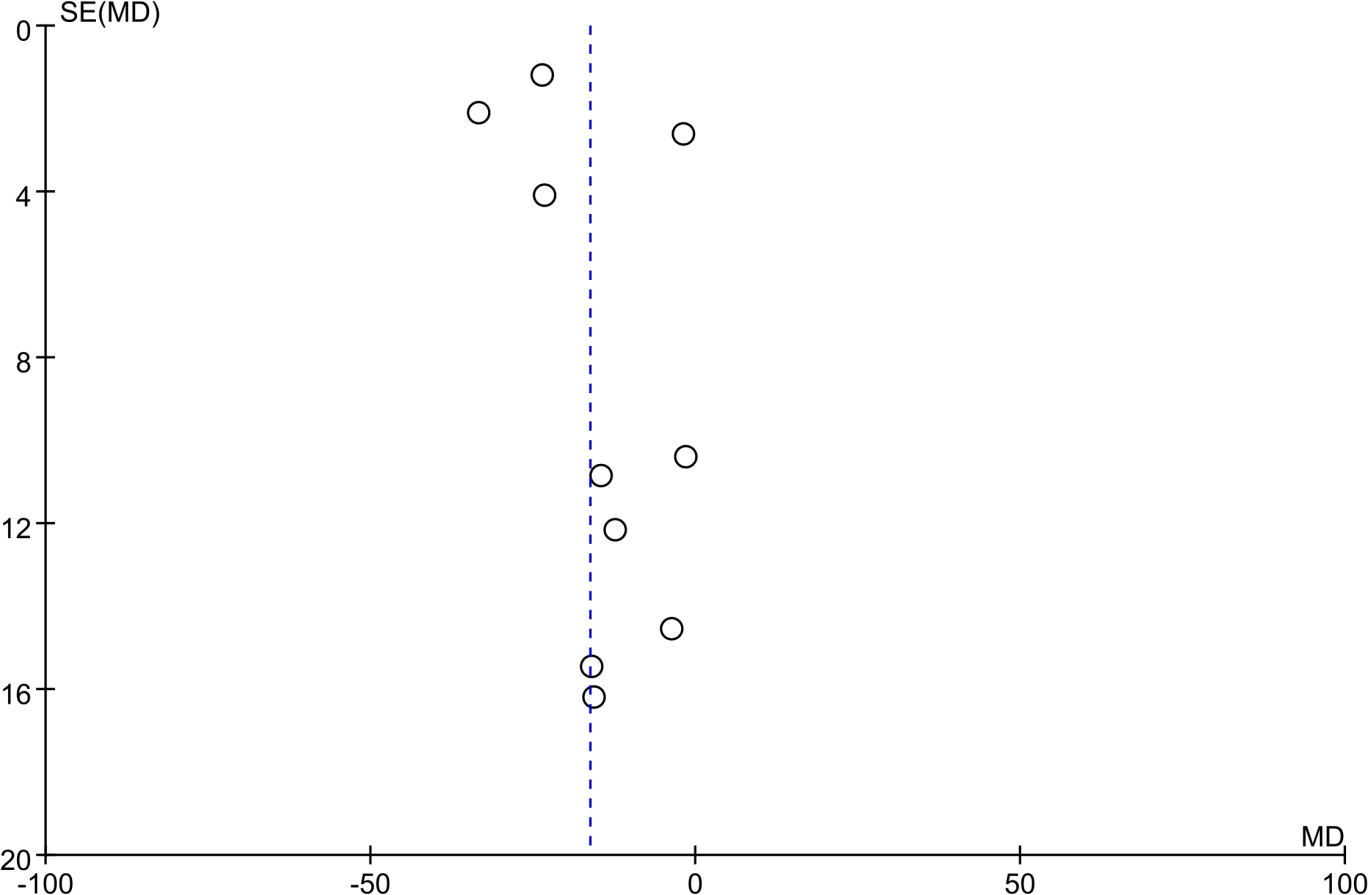
Funnel plot of VAT.

**Figure 5:**
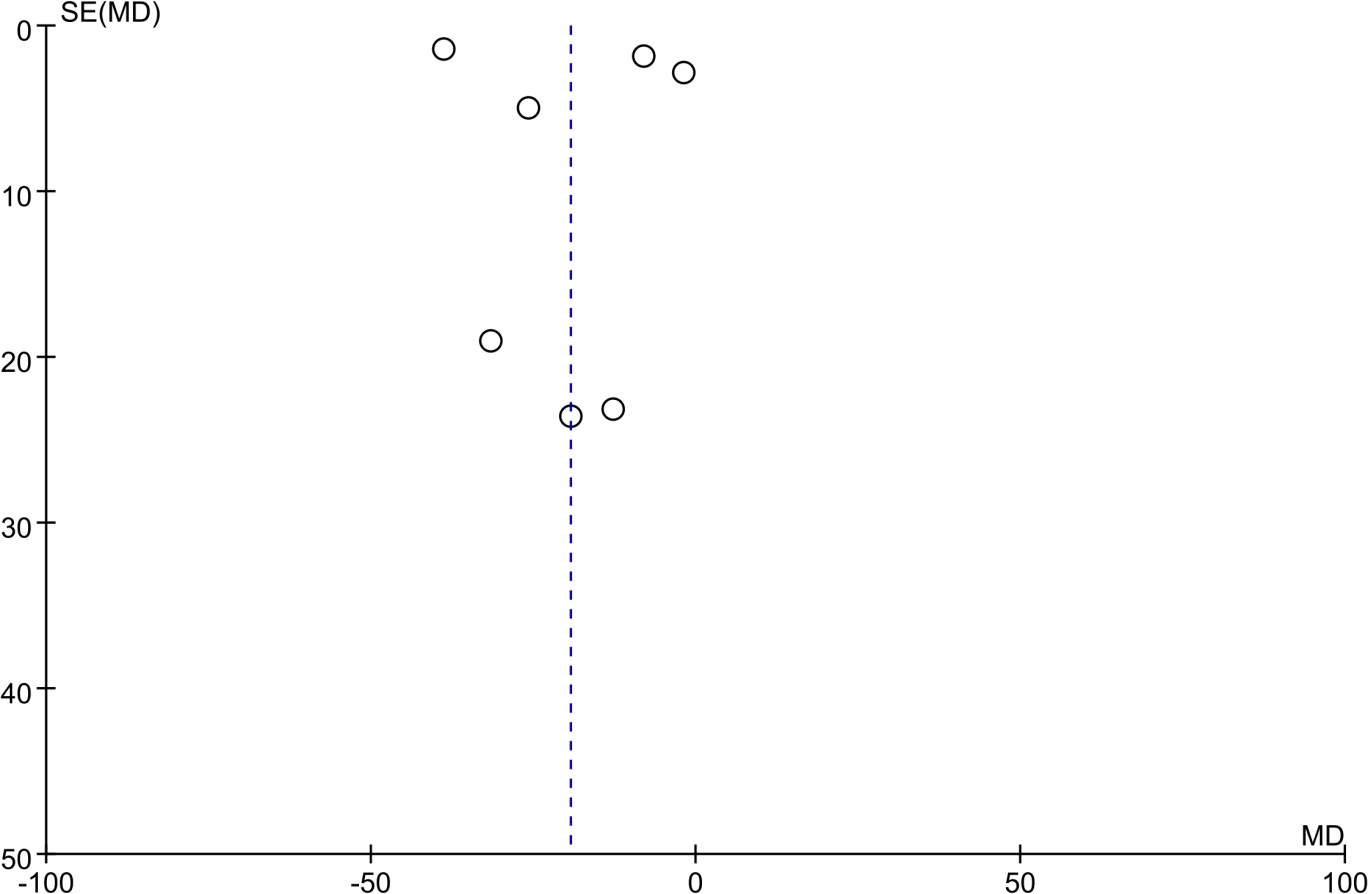
Funnel plot of SAT.

All studies reported results for VAT and 7 studies reported results for SAT. Meta-analysis results showed that compared with the control group, VAT (WMD = -16.29 cm^2^, 95% CI: -25.07 ~ -7.50, *P*<0.00001) and SAT (WMD = -19.34 cm^2^, 95% CI: -36.27 ~ -2.41, *P*<0.00001) in the SGLT2 inhibitors treatment group were significantly reduced (**Figures 2**, **3**). We observed a high degree of heterogeneity in both VAT (*P*<0.00001, I^2^ = 91%) and SAT (*P*<0.00001, I^2^ = 97%). Funnel plot results for both VAT and SAT showed that the scatter points corresponding to each study was largely distributed on the midline or largely symmetrically distributed, with no significant publication bias in either outcome, as shown in **Figures 4**, **5**.

Considering the high heterogeneity of VAT and SAT, we further conducted sensitivity analysis and subgroup analysis (**Table 2**). In the sensitivity analysis, removing any of the included studies caused no significant change in the results of VAT and SAT. Subsequently, subgroup analyses were performed based on the type of SGLT2, baseline BMI level, and duration of follow-up. The results showed that ipragliflozin significantly reduced VAT and SAT, while neither empagliflozin nor dapagliflozin reached statistical significance. As compared to the subgroup with BMI≥28 kg/m^2^, treatment with SGLT2 resulted in a more significant reduction in SAT (WMD = -25.17 cm^2^, 95% CI: -34.32 ~ -16.02, *P*<0.00001) in the subgroup with BMI<28 kg/m^2^. In addition, neither VAT nor SAT reached statistical significance in the subgroup with < 24 weeks of follow-up (*P*>0.05).

**Table 2:** Subgroup analysis.

Study characteristics	VAT (cm^2^)				SAT (cm^2^)			
	Study number	Patient number	WMD [95% Cl]	*P*-value	Study number	Patient number	WMD [95% Cl]	*P*-value
Empagliflozin	4	223	-2.55 [-7.47, 2.37]	0.31	1	51	-1.90 [-7.49, 3.69]	0.50
Dapagliflozin	2	99	-10.01 [-27.05, 7.04]	0.25	2	99	-23.87 [-52.72, 4.98]	0.10
Ipragliflozin	4	208	-26.38 [-33.20, -19.57]	0.0008	4	208	-23.42 [-44.82, -2.03]	0.03
BMI ≥ 28(kg/m^2^)	5	284	-18.22 [-30.67, -5.76]	0.004	3	161	-16.24 [-40.27, 7.80]	0.19
BMI < 28(kg/m^2^)	5	246	-16.78 [-25.85, -7.71]	0.0003	4	197	-25.17 [-34.32, -16.02]	<0.00001
Follow-up ≥ 24weeks	7	368	-17.65 [-27.40, -7.90]	0.0004	6	296	-19.37 [-37.08, -1.67]	0.03
Follow-up <24weeks	3	162	-11.34 [-28.70, 6.01]	0.2	1	62	-19.00 [-65.20, 27.20]	0.42

#### 3.4.2 Body weight and BMI

7 trials reported body weight, with high heterogeneity among the studies (*P*<0.00001, I^2^ = 88%). Meta-analysis results showed that as compared to the control group, the body weight (WMD = -2.36 kg, 95% CI: -2.89 ~ -1.83, *P*<0.00001) of patients in the SGLT2 inhibitor treatment group was significantly reduced. Only 4 trials reported BMI, and no heterogeneity existed among the studies (*P* = 0.84, I^2^ = 0%). The results also indicated that relative to the control group, patients in the SGLT2 inhibitor treated group had a significantly lower BMI (WMD = -0.8 kg/m^2^, 95% CI: -0.91 ~ -0.69, *P*<0.00001) (**Table 3**).

**Table 3:** Meta-analysis results of body weight, BMI and blood lipids.

Study characteristics	Study number	Patient number	WMD [95% Cl]	*P*-value
Body weight (kg)	7	381	-2.36 [-2.89, -1.83]	<0.00001
BMI (kg/m^2^)	4	222	-0.80 [-0.91, -0.69]	<0.00001
Total cholesterol (mg/dl)	7	391	-2.51 [-10.69, 5.67]	0.55
Triglyceride (mg/dl)	7	397	-24.41 [-45.79, -3.03]	0.03
LDL-C (mg/dl)	9	497	-1.31 [-9.97, 7.36]	0.77
HDL-C (mg/dl)	8	446	1.63 [0.62, 2.64]	0.002

#### 3.4.3 Blood lipids

According to the meta-analysis, triglycerides (WMD = -24.41 mg/dl, 95% CI: -45.79 ~ -3.03, *P* = 0.03) were significantly lower and HDL cholesterol (WMD = 1.63 mg/dl, 95% CI: 0.62 ~ 2.64, *P* = 0.002) was significantly higher in SGLT2 inhibitor-treated patients as compared to controls, while total cholesterol and LDL cholesterol failed to reach statistical significance (**Table 3**).

## 4 Discussion

In the meta-analysis involving 10 publications and 551 patients, we demonstrated that SGLT2 inhibitors, in addition to significantly reducing body weight and triglycerides, considerably lowered VAT and SAT in patients with type 2 diabetes.

Subgroup analysis of SGLT2 inhibitors drug categories by VAT and SAT revealed that ipragliflozin led to significant reductions in VAT and SAT, whereas empagliflozin and dapagliflozin seemingly caused no significant changes in VAT and SAT in patients with type 2 diabetes.

Differences in the drugs themselves may be one possible reason for the result. Neeland et al. analyzed the effects of empagliflozin on total body and visceral adiposity in over 6000 type 2 diabetes patients with high cardiovascular risk, finding that the drug significantly reduced indices of visceral adiposity and total body fat (11), which is inconsistent with our research results, even if the outcome indicators are different. In the case of our meta-analysis, only two studies used dapagliflozin treatment. Therefore, more or large clinical trials are needed for demonstrating the effects of the two drugs on VAT and SAT. Differences in the methods of measuring the main outcome indicators may be another reason for heterogeneity. Studies of ipragliflozin have mainly used computed tomography (CT) scan to determine VAT and SAT, whereas studies pertaining to empagliflozin and dapagliflozin have measured VAT and SAT by magnetic resonance imaging (MRI), DEXA, BIA.

We found that SGLT2 inhibitors treatment significantly reduced VAT and SAT when the follow-up time was longer than 6 months, whereas this positive result was not observed in trials with follow-up period of less than 6 months. A study indicated that the weight loss caused by SGLT2 inhibitors treatment was observed in the first week, and reached a stable level after 6 months (25). In our study, patients in all trials with follow-up time less than 6 months received a 3-month treatment, and such short treatment time may lead to the negative results.

Consistent with the results of several studies, we demonstrated that SGLT2 inhibitors considerably lower triglyceride levels in patients with type 2 diabetes (26, 27). At present, much controversy exists regarding the effect of SGLT2 inhibitor treatment on serum HDL-C and LDL-C levels. Calapkulu et al. found a decrease of 13.4 mg/dL in the LDL-C level in type 2 diabetes patients who had taken dapagliflozin (10mg/d) for 6 months (26). However, a study by Rau et al. showed that after 3 months of treatment for patients with type 2 diabetes, empagliflozin (10mg/d) led to an increase of 9 mg/dL in LDL-C (28). This growth is likely to be due to increased lipoprotein-lipase activity and delayed LDL turnover in the circulation (29). Therefore, further research is needed to explore the effect of SGLT2 inhibitor on blood lipids.

In healthy populations, normal adipose tissue has such functions as protecting internal organs, storing energy, producing heat and reducing inflammation. Obesity, insulin resistance, diabetes and inflammation are all associated with the phenotype and biological changes of adipose tissue (30). In obesity and diabetes, adipose tissue is infiltrated by increasing numbers of inflammatory and immune cells, losing its own homeostatic function and developing chronic low-grade inflammation (31). Dysfunctional adipose tissue reduces the release of protective factors (e.g. lipocalin, nitric oxide or protective prostaglandins) and increased activation of stress-related pathways, leading to the release of pathological adipokines (e.g. resistin, visceral adiponectin and leptin) and the development of low-grade inflammation, and promoting metabolic and cardiovascular dysfunction (32). Dysfunctional adipose tissue may also increase the production of tissue factors and bioactive isoprostanes, damage the sensitivity of platelets to insulin signals, enhance coagulation and impair fibrinolysis, and promote thrombosis (33). Nesti L et al. showed that in type 2 diabetic patients with normal cardiac function, higher epicardial adipose tissue thickness was associated with lower peak oxygen uptake, reduced systolic reserve, higher natriuretic peptide levels, as well as chronotropic insufficiency and impaired heart rate recovery (34). Epicardial fat also causes a mechanical constriction of the diastolic filling leading to micro-circulatory dysfunction, which, together with the pro-inflammatory effect and fibrosis of the underlying myocardium, impairs the relaxability of the left ventricle and increases its filling pressure (35).

Contributing to reduced VAT and SAT, SGLT2 inhibitors also reduce ectopic adipose tissues, such as epicardial adipose tissue (36), hepatic adipose tissue (37) and perivascular adipose tissue (38), without affecting muscle mass and bone mineral content (9, 39). Sakurai et al. found that SGLT2 inhibitors reduced plasminogen activator inhibitor-1 (PAI-1) along with VAT, improved fibrinolysis, and increased serum high molecular weight adiponectin (17). In type 2 diabetes patients with non-alcoholic fatty liver disease, SGLT2 inhibitors can improve liver steatosis and fibrosis (18, 20), lowering serum aspartate aminotransferase (AST) and alanine aminotransferase (ALT) levels (37).

The mechanism by which SGLT2 inhibitors reduce adipose tissue is not completely clear. An animal study found that SGLT2 inhibitors triggered glycogen depletion signal and actuated liver-brain-adipose axis, promoting lipolysis (40). Lauritsen et al. showed that SGLT2 inhibition reduced GLUT4 gene and protein expression in adipose tissue, possibly by reducing glycerol formation and rebalancing substrate utilization away from glucose oxidation and lipid storage capacity (41). Moreover, SGLT2 inhibitors increase fat utilization by activating M2 macrophages (42). Undoubtedly, the role of SGLT2 inhibitors in ameliorating cardiovascular risk relates not only to the adipose tissue reduction, but, to their pleiotropic effect in reducing multiple risk factors (43).

There are some limitations to our study that should be noted. First, we included a limited number of trials and sample sizes, and thus more RCTs are needed to further validate the current results. Secondly, although all of these included studies were RCTs, seven of the ten included studies were not double-blinded, increasing the risk of performance bias. Third, the majority of RCTs included in the study were from Asia, so ethnic differences and Asian-specific lifestyles may have affected the results. Fourth, most trials included in the study had a short follow-up period, except one study that had a 26-month follow-up time. Fifth, significant heterogeneity was noted in the analysis of both the VAT and SAT, which may be due to different demographic characteristics and substantial differences in intervention type and design. Sixthly, another limitation is the difference of therapeutic drugs and doses used in each study, as well as various side effects caused by other drugs used in the trial population.

## 5 Conclusion

Our pooled results show that SGLT2 inhibitors significantly reduce VAT, SAT, body weight, and triglycerides in type 2 diabetes patients, which may correlate with the cardiovascular protective effects of the inhibitors and suggest a new therapeutic strategy for obese patients. Due to the limited sample size of the study and high heterogeneity of the included studies, the effects of SGLT2 inhibitors on adipose tissue need to be further investigated by resorting to a large sample of long-term RCTs.

## Data availability statement

The original contributions presented in the study are included in the article/**Supplementary Material**. Further inquiries can be directed to the corresponding author.

## Author contributions

XL and TL designed the study. XL and LC collected the data. YC performed the meta-analysis and drafted the manuscript. YC, XY, and CM partially planned the research. XL edited the manuscript. All authors contributed to the article and approved the submitted version.
