# Supplementary material for: The effects of Sodium-glucose cotransporter 2 inhibitors on adipose tissue in patients with type 2 diabetes: A meta-analysis of randomized controlled trials
Source: Front Endocrinol (Lausanne). 2023 Jan 27;14:1115321. doi: 10.3389/fendo.2023.1115321 (PMC9911550; doi:10.3389/fendo.2023.1115321)
Supplement: Supplementary file 1 [file Table_1.docx]

Supplementary Material

**The effects of Sodium-glucose cotransporter 2 inhibitors on adipose tissue in patients with type 2 diabetes: a meta-analysis of randomized controlled trials**

Xindong Liu, Ying Chen, Tao Liu*, Ling Cai, Xiaofeng Yang, Chuan Mou

*** Correspondence:** Tao Liu: nclt456@sina.com

# Supplementary Tables **1 PubMed search strategy.**

| #1 | Diabetes Mellitus, Type 2[MeSH Terms] |
| --- | --- |
| #2 | Type 2 Diabetes Mellitus[Title/Abstract] OR Type 2 Diabetes[Title/Abstract] OR T2DM[Title/Abstract] |
| #3 | #1 OR #2 |
| #4 | [Sodium-Glucose Transporter 2 Inhibitors](https://www.ncbi.nlm.nih.gov/mesh/2027927)[MeSH Terms] |
| #5 | SGLT2 Inhibitors[Title/Abstract] OR Empagliflozin[Title/Abstract] OR Dapagliflozin[Title/Abstract] OR Canagliflozin[Title/Abstract] OR Ertugliflozin[Title/Abstract] OR Sotagliflozin[Title/Abstract] OR Velagliflozin[Title/Abstract] OR Ipragliflozin[Title/Abstract] |
| #6 | #4 OR #5 |
| #7 | Adipose Tissue[MeSH Terms] |
| #8 | Fatty Tissue[Title/Abstract] OR Fat Pad[Title/Abstract] OR Body Fat[Title/Abstract] OR Ectopic Adipose Tissue[Title/Abstract] OR Ectopic Fat[Title/Abstract] OR Visceral Adipose Tissue[Title/Abstract] OR Visceral Fat[Title/Abstract] OR Subcutaneous Adipose Tissue[Title/Abstract] OR Subcutaneous Fat[Title/Abstract] |
| #9 | #7 OR #8 |
| #10 | #3 AND #6 AND #9 |

# Su**pplementary Tables 2 Changes of outcome indicators before and after treatment.**

| **Control** | **HDL-C (mg/dl)** | / | 3 ± 10 | 5 ± 1.4 | -1 ± 1.1 | 1.7 ± 11.6 | -0.7 ± 1 | 2.2 ± 15.5 | 2.1 ± 8.9 | / | 2.3 ± 8.5 |
| --- | --- | --- | --- | --- | --- | --- | --- | --- | --- | --- | --- |
|  | **LDL-C (mg/dl)** | / | 10 ± 29.1 | 10.5 ± 3.5 | 0.7 ± 17.9 | 6.9 ± 23.6 | -9.3 ± 3.7 | 0.9 ± 25.5 | 0.9 ± 5.9 | 0 ± 27.2 | -8.1 ± 17.8 |
|  | **Triglyceride (mg/dl)** | / | 13 ± 34.6 | -22.8 ± 18.7 | 32.3 ± 36.1 | -3.1 ± 88.8 | 11 ± 13.9 | / | -16.7 ± 110.7 | / | 4.4 ± 40.7 |
|  | **Total cholesterol (mg/dl)** | / | 13 ± 34.6 | 8.9 ± 5.5 | 3.6 ± 4.2 | / | -11.9 ± 5.8 | / | 3.6 ± 40.3 | -4 ± 37.7 | -5.8 ± 21.3 |
|  | **BMI (kg/m^2^)** | / | / | / | / | -0.1 ± 3.6 | 0.2 ± 0.2 | -1.4 ± 6.3 | -0.1 ± 4.6 | / | / |
|  | **Body weight (kg)** | / | 0.45 ± 0.77 | 0.9 ± 0.4 | -0.22 ± 0.38 | / | 0.4 ± 0.6 | -4 ± 16.5 | -0.3 ± 12.9 | / | -0.68 ± 1.75 |
|  | **SAT(cm^2^)** | 34.4 ± 10.7 | 13 ± 92.1 | 15.7 ± 6 | 6.7 ± 14.1 | 1.3 ± 87.2 | -1.4 ± 5 | / | / | / | -3.5 ± 41.7 |
|  | **VAT(cm^2^)** | 17.7 ± 10 | 17 ± 57.2 | -2.6 ± 4.9 | 11.3 ± 11.5 | -5.7 ± 36.8 | 7 ± 7.7 | -6 ± 49.3 | 11.6 ± 58.4 | 3.6 ± 72.9 | −4.5 ± 34.3 |
|  |  |  |  |  |  |  |  |  |  |  |  |
| **Intervention** | **HDL-C (mg/dl)** | / | 2 ± 9.5 | 5.5 ± 1.5 | 1.4 ± 1.1 | 5.3 ± 13.8 | 1.7 ± 1.6 | 4.4 ± 18.2 | 2.7 ± 9.2 | / | 3.5 ± 5.8 |
|  | **LDL-C (mg/dl)** | / | -4 ± 28.8 | 1.9 ± 2.7 | -4.3 ± 19 | 1.3 ± 30.9 | 2.1 ± 2.7 | 3.6 ± 26.1 | -3.7 ± 14.7 | 1 ± 32.2 | 0.4 ± 13.1 |
|  | **Triglyceride (mg/dl)** | / | -12 ± 66.8 | -24.5 ± 10.3 | -57.6 ± 37.5 | -15.1 ± 51.8 | -10.8 ± 11.6 | / | -20.7 ± 140.3 | / | -8.9 ± 52.2 |
|  | **Total cholesterol (mg/dl)** | / | -4 ± 36.6 | 2 ± 3 | -8.4 ± 4.3 | / | -2.5 ± 3.6 | / | -2 ± 26.1 | 0 ± 41 | 3.9 ± 23.2 |
|  | **BMI (kg/m^2^)** | / | / | / | / | -0.7 ± 4.9 | -0.6 ± 0.1 | -0.6 ± 5.6 | -1 ± 3.2 | / | / |
|  | **Body weight (kg)** | / | -1.51 ± 1.28 | -2.3 ± 0.5 | -2.78 ± 0.4 | / | -1.6 ± 0.4 | -1.9 ± 18.9 | -2.7 ± 12.1 | / | -2.4 ± 2.13 |
|  | **SAT(cm^2^)** | 32.5 ± 9.2 | -6 ± 82.5 | -23 ± 6.2 | -18.9 ± 15.7 | -11.2 ± 85.7 | -9.3 ± 7.2 | / | / | / | -35 ± 77.8 |
|  | **VAT(cm^2^)** | 16 ± 8.4* | 1 ± 60 | -26.1 ± 4.9 | -12 ± 12.7 | -7.3 ± 41.2 | -26.2 ± 3.7 | -9.7 ± 48.6 | -0.6 ± 43.9 | -12.1 ± 35.6 | -19.0 ± 36.1 |
| **Study** | | Kim 2014 | Bando 2017 | Ito 2017 | Inoue 2019 | Shimizu 2019 | Han 2020 | Sakurai 2020 | Chehrehgosha 2021 | Gaborit 2021 | Horibe 2022 |
