# Supplementary figures and images for: The effects of Sodium-glucose cotransporter 2 inhibitors on adipose tissue in patients with type 2 diabetes: A meta-analysis of randomized controlled trials
Source: Front Endocrinol (Lausanne). 2023 Jan 27;14:1115321. doi: 10.3389/fendo.2023.1115321 (PMC9911550; doi:10.3389/fendo.2023.1115321)

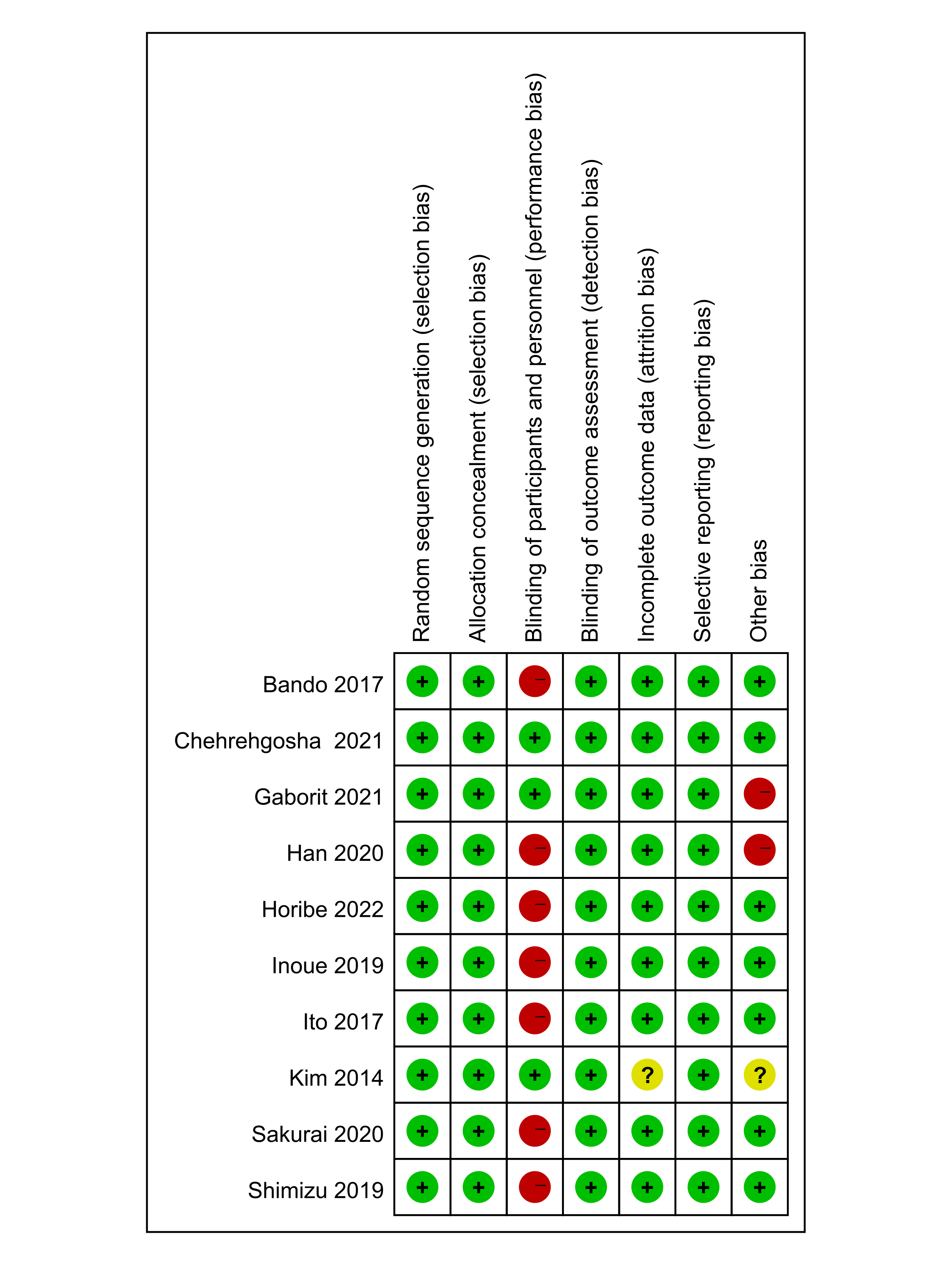

Supplement: Supplementary file 2 [file Image_1.tif]
